# Conflicts of interest of editors of medical journals

**DOI:** 10.1371/journal.pone.0197141

**Published:** 2018-05-18

**Authors:** Waqas Haque, Abu Minhajuddin, Arjun Gupta, Deepak Agrawal

**Affiliations:** 1 Department of Internal Medicine, University Texas Southwestern Medical Center, Dallas, TX, United States of America; 2 Department of Clinical Sciences, University Texas Southwestern Medical Center, Dallas, TX, United States of America; 3 Division of Gastroenterology, University Texas Southwestern Medical Center, Dallas, TX, United States of America; University of Illinois-Chicago, UNITED STATES

## Abstract

**Background:**

Almost all medical journals now require authors to publicly disclose conflicts of interests (COI). The same standard and scrutiny is rarely employed for the editors of the journals although COI may affect editorial decisions.

**Methods:**

We conducted a retrospective observational study to determine the prevalence and magnitude of financial relationships among editors of 60 influential US medical journals (10 each for internal medicine and five subspecialties: cardiology, gastroenterology, neurology, dermatology and allergy & immunology). Open Payments database was reviewed to determine the percentage of physician editors receiving payments and the nature and amount of these payments.

**Findings:**

703 unique physician editors were included in our analysis. 320/703 (46%) received 8659 general payments totaling $8,120,562. The median number of payments per editor was 9 (IQR 3–26) and the median amount per payment was $91 (IQR $21–441). The median total payment received by each editor in one year was $4,364 (IQR $319–23,143). 152 (48%) editors received payments more than $5,000 in a year, a threshold considered significant by the National Institutes of Health. COI policies for editors were available for 34/60 (57%) journals but only 7/34 (21%) publicly reported the disclosures and only 2 (3.%) reported the dollar amount received.

**Interpretation:**

A significant number of editors of internal medicine and subspecialty medical journals have financial COI and very few are publicly disclosed. Specialty journal editors have more COI compared to general medicine journal editors. Current policies for disclosing COI for editors are inconsistent and do not comply with the recommended standards.

## Introduction

Financial relationships between medical publishing personnel (investigators, authors, reviewers and editors) and biomedical industry are common[1, 2]. However, conflicts of interest (COI) can arise in situations when a physician’s or a researcher’s professional judgment concerning a primary interest is at risk of being biased by a secondary interest, resulting in possible harm to patients or the integrity of research. A COI, thus, refers to a situation in which there is a risk of bias and resulting harm, not a situation in which bias or harm necessarily occurs. The secondary interests can be financial, personal or professional [3]. Financial COI (FCOI) are not necessarily more corrupting than other secondary interests but relatively more objective, fungible, quantifiable and more effectively regulated [4].

Recognizing the importance of unbiased research, almost all medical journals now require authors to publicly disclose COI. The same standard and scrutiny is rarely employed for the editors of the journals [4]. Editors are the gatekeepers of information that gets published- they choose peer reviewers, accept or deny a manuscript, can influence the tenor of discussion and the timing of publication.

Multiple professional editors’ associations recommend that relevant COI of all editorial staff be known and that editors should recuse themselves from editorial decisions if they have potential conflicts related to articles under consideration[5–9]. The editorial process, by design, is not transparent and it is unclear if these standards are adhered to. Reports of biased publishing by editors with FCOIs, although rare, severely undermine the trust of readership (academics, health professionals and the public) in the scientific publication process.

There are very limited data on prevalence of FCOI of physician editors. One study based on voluntary disclosures of FCOI by physicians at scientific meetings reported that 29% of editors from five leading spine journals had FCOI [10]. There is a concern of underreporting with voluntary disclosures. The Open Payments program, a result of the 2010 Affordable Care Act, mandates all pharmaceutical and medical device manufacturers to publicly report payments of value to physicians[11]. We queried the searchable Open Payments database to determine the prevalence and magnitude of financial relationships among editors of medicine and subspecialty journals: cardiology, gastroenterology, neurology, dermatology and allergy & immunology. These specialties were chosen due to known significant financial ties with the biomedical industry [1, 10, 12].

## Methods

### Medical specialty and clinical journal selection

We chose to examine FCOI of editors of internal medicine and five subspecialties—cardiology, gastroenterology, neurology, dermatology and allergy & immunology. The top 9 journals were selected from each specialty based on the SCImago Journal Rank indicator and Google Scholar’s h5-index, and had to meet the following criteria–(1) have at least 5 editors and (2) at least 25% of the editors should be physicians licensed to practice in the US. We excluded journals with disease-specific titles since comparing editors of journals of certain diseases with more expensive drugs (eg- molecular agents in inflammatory bowel disease) would not allow a fair comparison. We also included editors of each subspecialty listed on the website UptoDate (http://www.uptodate.com) since it is widely subscribed to with an estimated 200 million topic views per year [13]. We thus analyzed 10 journals within each specialty. Surgical specialties were excluded due to the greater emphasis on medical devices compared to drugs, which can alter the context and form of industry relationships.

Names of the editors were obtained by reviewing the websites of each journal and confirmed with print versions of each journal from January 2014 and January 2015. The editors of each journal were categorized based on their roles and responsibilities: 1) Chief Editor, assumed to have the final responsibility for publications and compliance with the policies of a journal; 2) Associate editors (including Associate, Assistant and Executive editors), who manage the review process and often content experts in a specific field. Editors listed as physicians in the United States were then queried on the CMS Open Payments program website. The profiles were matched using full name, listed specialty, location of medical practice (city, state and zip code) and state medical licensure. These were analyzed separately since in many journals, only chief editors have decision-making power. S1 Dataset contains the underlying data set and the individual data elements.

**Fig 1:**
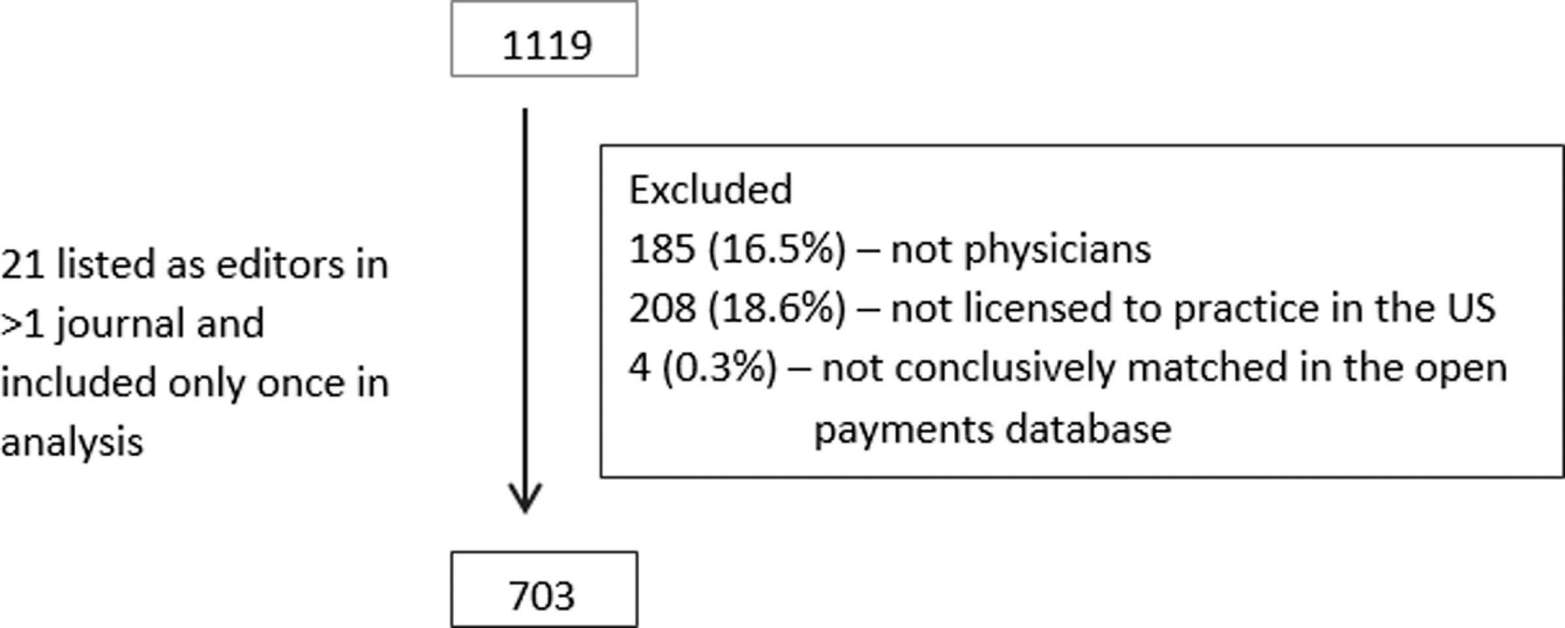
Flowchart describing the inclusion of editors in the study.

A total of 1119 editors (1086 editors from 60 journals, and 33 editors from UpToDate) were identified. The following editors were excluded from analysis: 185 (16.5%) who were not physicians, 208 (18.6%) not licensed to practice medicine in the United States and 4 (0.3%) editors who could not be conclusively matched on Open Payments database. Furthermore, 21 physicians were listed as editors for two journals (Fig 1).

### Industry payments data collection

The Open Payments database was accessed in July 2016 and information from the year 2015 was collected [11]. The database classifies payments as: (1) general payments which includes consulting fees, royalties, travel, lodging, food and beverage, and (2) research payments, which includes research funding and associated payments. For this study we included only general payments in the analysis since they are more closely associated with the drug promotion. Furthermore, the research payments may include amounts not directly paid to the physicians but for conducting a study. We also searched each editor’s name on the ProPublica Dollars for Docs website [14], which analyzes the CMS Open Payments source data and categorizes information on general payments by specific drugs or device.

### Outcomes measures and statistical analysis

The outcomes of interest were (1) percentage of physician editors receiving payments, (2) the nature and distribution of payments, and (3) the most common drugs or devices associated with payments. The payments to editors were analyzed for each medical specialty and editorial role. The payments did not follow a normal distribution for either editors or journals and were therefore reported as median values. Comparison between different journals in terms of payments received was performed using Kruskal-Wallis Analyses of Variance (ANOVA). Statistical significance level was set at 0.05.

## Results

A total of 703 unique editors were included in our analysis of overall payments to editors. The number of editors in each journal and percentage of editors receiving general payments is given in Table 1.

**Table 1:** The number of editors in each specialty, and percentage of editors receiving general payments.

Specialty	Number of Editors in each specialty	Median (interquartile range) number of Editors in each Journal	Number of US Physician Editors Included in Analysis	Number (Percent) of Physician Editors receiving Payments
Allergy	123	9.5 (8–14)	64	30 (46%)
Cardiology	180	16 (9.75–26)	133	84 (63%)
Dermatology	249	14.5 (9.5–23.5)	136	46 (33%)
Gastroenterology	184	14.5 (10.5–20.75)	136	94 (69%)
Internal Medicine	153	15.5 (11.25–19)	134	23 (17%)
Neurology	230	13 (9.25–24.75)	100	43 (43%)

### Payments to physician editors of all journals

Of the 703 individual editors who satisfied inclusion and exclusion criteria, 320/703 (46%) received 8659 general payments totaling $8,120,562. The median number of payments per editor was 9 (IQR 3–26) and the median amount per payment was $91 (IQR $21–441). The median total payment received by each editor in one year was $4,364 (IQR $319–23,143). The numbers reported here are for the editors who got paid according to open payments database.

The type of general payments made to editors in each payment category is shown in Table 2. Of the editors receiving payments, the most number of payments were for food and beverage, with 287 (90%) editors receiving 4334 payments (median value $351 and total amount $214,827). The most amount of payments were for consultation with 183 (57%) editors receiving 1613 payments (median value $11,340 and total amount $6,363,952). There were 121 (38%) editors who received more than $10,000 in consulting payments in a year.

**Table 2:** The type of general payments made to editors in each payment category.

Payment Type	Number of Payments	Payment Per Editor		
		Total Amount ($)	Median Amount ($)	Interquartile Range ($)
Consulting Fee	1616	6,363,952	11340	4100, 33692
Education	144	79370	72	15, 891
Food and Beverage	4432	214,827	351	101, 929
Grant	18	41,322	3000	200, 5000
Honoraria	113	113	3100	2000,6500
Royalty or License	7	25260	4998	263, 20000
Travel and Lodging	2329	1,147,097	2625	883, 9141

### Payments to physician editors by specialty

**Fig 2:**
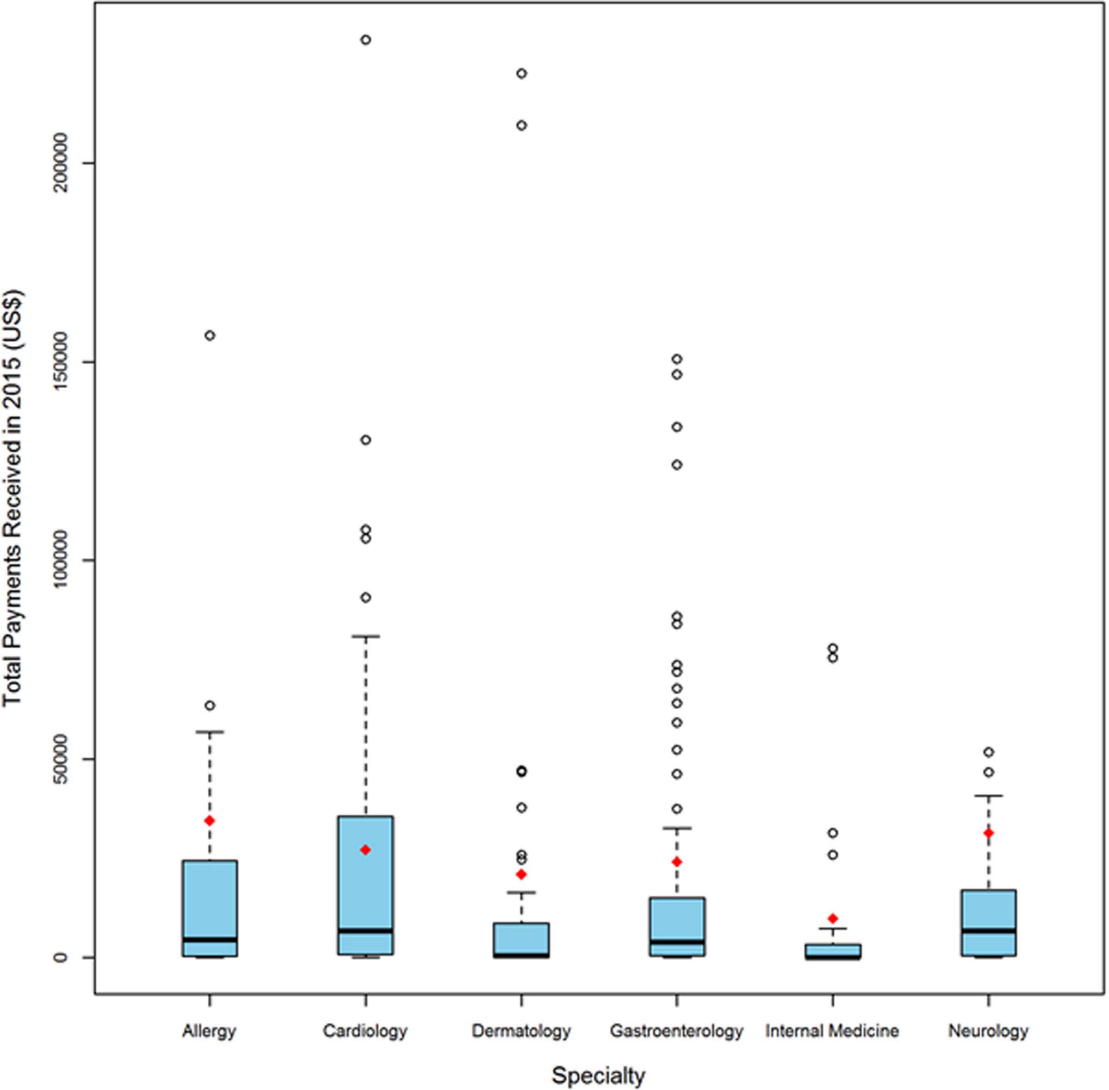
Total Payments received by editors in each specialty. Blue columns–Chief editors; Red diamond-mean; Black line–median.

The number and percentage as well as mean, median and interquartile range of the total payment received by an editor of each specialty journal is shown in Table 3. The editors of cardiology journals (86%) followed by gastroenterology (84%) were most likely to receive payments. The percentage of editors receiving more than $10,000 ranged from 17–45% with highest for cardiology and lowest for internal medicine. Fig 2 shows the number of editors receiving payments above the 75^th^ percentile in each specialty, and this was most common in gastroenterology.

**Table 3:** The number and percentage as well as mean, median and interquartile range of the total payment received by an editor of each specialty journal.

Physician Specialty	Median (interquartile range)	Mean (standard deviation)	Total Payments	Number of Editors who Received Payments	
				> $5,000	> $10,000
Allergy	4506 (394, 31590)	34510	1035299	14 (47%)	11 (37%)
Cardiology	6841 (846, 36235)	27113	2277515	47 (56%)	38 (45%)
Dermatology	537 (106, 8683)	20976	964901	15 (33%)	11 (24%)
Gastroenterology	4831 (478, 17144)	24120	2267237	47 (50%)	38 (40%)
Internal Medicine	103 (27, 5008)	9872	227046	6 (26%)	4 (17%)
Neurology	7354 (491, 20040)	31362	1348563	23 (54%)	18 (42%)

### Payments to physician editors by their editorial role

**Fig 3:**
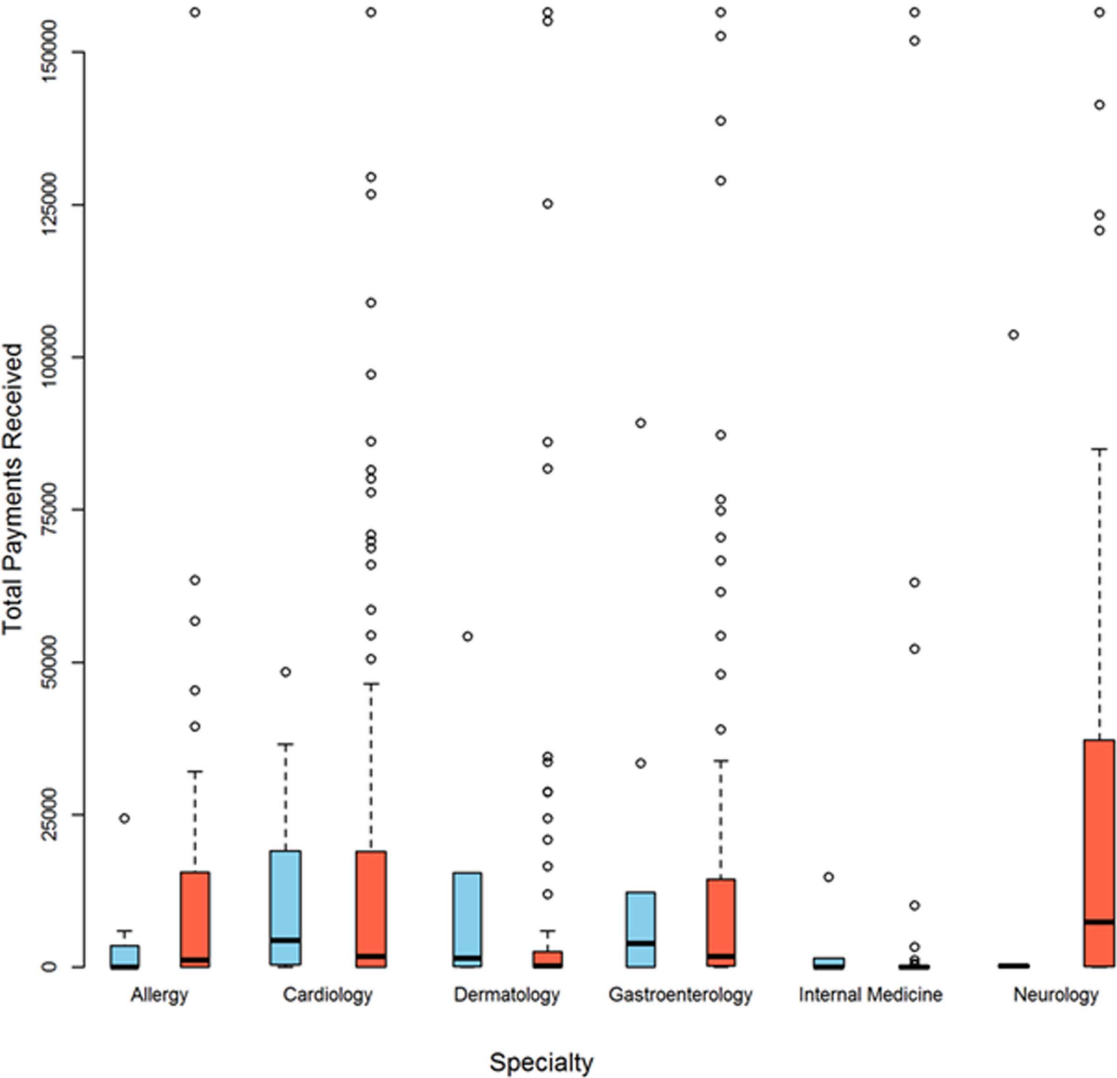
Total Payments received by the chief editors and associate editors in each specialty. Blue columns–Chief editors; Red Columns- Associate Editors; Black line-median.

Overall, 31 (9.7%) Chief Editors received payments. Between specialties, 8.3% chief editors of cardiology received payments compared to 17.4% chief editors of internal medicine journals. Median and mean payments received by Chief editors and Associate editors for each specialty are given in Table 4. For all journals taken together, there was no significant difference in median payments received by the Chief editors and Associate editors (p = 0.31). Fig 3 shows that a number of associate editors in all specialties received payments more than 75^th^ percentile, with many receiving in excess of $50,000.

**Table 4:** Median and mean payments received by Chief editors and Associate editors for each specialty.

Physician Specialty	Chief Editors receiving payments		Associate Editors receiving payments	
	Number (%)	Median Payment (interquartile range)	Number (%)	Median Payment (interquartile range)
Allergy	5 (16.7)	1009 (39, 5971)	25 (83.3)	4534 (847, 32058)
Cardiology	7 (8.3)	6952 (2457, 30377)	77 (91.7)	6730 (763, 36748)
Dermatology	4 (8.7)	2550 (233, 10496)	42 (91.3)	532 (106, 8683)
Gastroenterology	8 (8.5)	7986 (1184, 21936)	86 (91.5)	3887 (478, 17144)
Internal Medicine	4 (17.4)	385 (18, 4050)	19 (82.6)	103 (33, 5008)
Neurology	3 (7)	112 (79, 34250)	40 (93)	7994 (842, 18482)

### Products associated with payments

The products most commonly associated with payments to physicians were Rifaximin ($247,886), Naloxegol ($156,202), Ticagrelor ($138,270), Tofacitinib ($133,010) and Droxidopa ($116,598).

### Policies and public disclosure of FCOIs of editors

COI policies for editors were publicly (website or print) available or obtained via correspondence with the respective editorial office for 34/60 (57%) journals. Of the 34 journals with a COI policy for editors, 26 (76%) required specific disclosures but only 7 (21%) publicly reported the disclosures and only 2 (3.3%) reported the dollar amount received.

## Discussion

Our analysis of 2015 Open Payments data shows that financial relationships between biomedical industry and editors are common with almost half of the editors having received payments. The median number of payments received by each editor in a year was 9, indicating frequent interactions of editors with the industry, which can influence physician behavior independent of amount of payment. However, despite recommendations that potential, relevant or significant FCOIs of editors be disclosed, the details are seldom available.

In a recent study, Liu, et al. evaluated payments to editors of 26 medical specialties using the 2014 Open Payments database.[15] Like our study, they found that half the editors received some form of payment. Interestingly, they also reported that median general payments to editors of a specific specialty were significantly higher than reported median general payments to all physicians in that specialty. For example, median payments to cardiology journal editors was $2664, compared with median payments of $582 to all cardiologists. However, their study included only 2 journals per specialty (total 52 journals), limiting number of editors per specialty. As an example, there were only 5 eligible urology editors and 9 eligible surgery editors who were included in their study. It also raises questions if the two journals were representative of that specialty. We performed a more comprehensive review of each specialty, and the minimum number of eligible editors in a specialty we evaluated was 123 (allergy), allowing us to draw more accurate conclusions about generalizability of the data to journals in the specialty. Additionally, we did not include surgical journals or pathology journals, since direct comparisons between vastly different specialties are difficult. As an example, in the study by Liu, et al., pathology editors received mean annual payments of $11, compared with $92,828 for orthopedics editors.[15] They had also included some journals that do not publish original research. Apart from analyzing more recent (2015) data, we also analyzed chief editors and associate editors separately, and did not find a statistically significant difference in payment amounts between them.​

These results raise several questions. What is *potential* and what is *relevant* in the context of an FCOI? Is there a monetary amount that makes a financial interest *significant*? Who decides? What is the intended benefit of public disclosure of FCOIs? Even if the FCOIs are disclosed, how do we know that appropriate remedial actions are being followed and enforced?

‘Potential’ and ‘relevant’ are subjective terms open to interpretation. McCoy and Emanuel recently opined that there are no “potential’ COI and “the notion of a potential COI reflects the mistaken view that a COI exists only when bias or harm actually occurs”. Who should decide? Is the “potential” COI limited to the product or to the company that makes the product? For example, one cardiology journal editor received more than $50,000 in consulting fees for apixaban, an oral anticoagulant, in a single year. Should this editor recuse himself or herself from editorial decisions on all manuscripts related to apixaban or all oral anticoagulants or all cardiology medications marketed by that company (which would include many antihypertensive and lipid lowering medications)? A broad definition of “potential’ would include the latter, and the editor would be unable to participate in editorial decisions for most submissions to the journal. Policies regarding what defines a COI, especially potential COI, need to be detailed.

Furthermore, is there a monetary amount that makes the FCOI *significant*? It is generally accepted that the greater the financial interest, the greater the likelihood professional judgment will be biased, and the more severe the COI. One study showed that brand-name prescribing of statins increased when physicians received $2000 or more from related companies [16]. The United States Department of Health and Human Services mandates disclosure of FCOIs more than $5000. Notably, the reporting threshold was lowered from $10,000 in 2011 to ensure greater scientific objectivity and integrity. In our study, the median amount received by each editor was close to $5,000 and 38% received >$10,000, indicating “significant” FCOI.

However, much smaller amounts can influence physician behavior. In a recent study, even receipt of less than $20 meal by physicians was associated with an increased rate of prescribing the brand-name medications that were being promoted [17]. Behavioral research shows that such actions by physicians are often made on a subconscious level [18, 19]. Moore and Loewenstein, in their analysis of the psychology of COI, argue that “the automatic nature of self-interest gives it a primal power to influence judgment and makes it difficult for people to understand its influence on their judgment, let alone eradicate its influence” [20].

Sometimes decisions by editors with FCOI are not subconscious but appear to be overtly biased. For example, an orthopedic surgeon, during his tenure as an editor published many studies in his journal favoring products from a company, which paid him millions of dollars in patent royalties [21]. In another report, an editor co-authored a favorable review of vagus nerve stimulation therapy for treatment of depression and published in his journal, while he was a paid consultant for that product. The FCOI were not disclosed [22, 23]. In 1995, Mayor et al. published results of a randomized study showing synthroid was “therapeutically inequivalent” to generic thyroxine preparations. Mayor was an employee of the company that made synthroid and also the associate editor of the journal where the article was published [24]. The same data published two years later—in another journal—reported very different conclusions [25]. In 2011, a prominent child psychiatrist was disciplined for failing to disclose $1.4 million in personal payments from the makers of antipsychotics, a drug category he promoted for bipolar disorder in children [26]. During this time, he was also on the editorial board of 7 peer-reviewed psychiatry journals [27]. Instances such as these maybe rare but sow the seeds of doubt and mistrust among public. It is not surprising, then, that despite remarkable gains in research, public trust in science in the last 40 years has remained less than 50% [28].

One way to gain trust is by encouraging transparency of FCOI, as recommended by the professional editors’ organizations [9] and supported by the editors [29]. A survey by Haivas et al, showed that 63% of editors of medical journals supported declaration of editors’ FCOI [29]. Still, in our study the basic FCOI information of editors was available for only 12% editors. Our results are similar to a study in 2013, which reported that only 40% of the 399 highest-impact biomedical journals required editors to disclose their COIs [30]. The premise of public disclosures of FCOIs by the editors is that readers will have an opportunity to question worrisome influences. Furthermore, disclosures have a self-calibrating quality–a small amount such as a free meal may seem inconsequential but would not be worth the hassle of disclosing. On the other hand, consulting fees of thousands of dollars may be more tempting but also have more serious repercussions. In either situation, FCOIs would be best avoided [31]. For disclosures to be effective, few criteria should be followed. *First*, the FCOIs should be consistently and truthfully declared. Research has shown that FCOIs among physicians are often underreported [4, 13, 32–35]. *Second*, for readers to make any meaningful conclusions, the financial disclosures should be specific and include information on type and magnitude of FCOIs. A disclosure statement such as “has received consulting and/or speaker fees from a company” is quite unhelpful. That company could be making hundreds of devices and drugs, and the payments could range from hundreds to thousands of dollars. In our study, the specifics and dollar amounts received by the editors were reported by only 2 journals. *Third*, the disclosures should be publicly and readily accessible. We were unable to get details about individual editors’ FCOIs for nearly 90% of journals after reviewing their websites, printed edition and after contacting the journals’ editorial office.

Even if all the disclosure etiquettes are followed, it can be quite difficult to completely discount the ill effects of FCOIs. Only a percentage of readers may review the disclosed FCOIs, few would know how to interpret the information and even fewer may soundly overcome their own anchoring and inherent biases. It is even argued that disclosing FCOIs can have unintended consequences and perverse effects. First, those declaring a FCOI may feel morally licensed to deviate from the norms of objectivity because they have declared a COI [36]. Second, a person with FCOIs may be viewed as more knowledgeable or expert in the field and thus their decisions may be viewed more favorably than warranted[36].

Given these issues and inherent skepticism about financial relationships, one may ask–is it necessary for editors to have any FCOIs? Are a few thousand dollars received by editors worth the reputation of the journal for unbiased publishing? Some journals do not think so and hold a policy that “none of the editors should have any financial relationship with any biomedical company”[35]. Others argue that a “purist” approach, whereby an editor has no potential COI, would lower the quality of the editorial process since there would be few qualified physician editors interested in the job. Our analysis shows that this is not true since half the editors had no FCOIs. At the very least, the Chief editors of medical journals should have no FCOIs [4]. The Institute of Medicine has a similar viewpoint stating that all chairs and co-chairs of guidelines committees should not have any FCOIs [37].

Our study has limitations. *First*, there is no information available if editors receiving industry payments participated in any related editorial decisions. The journals do not disclose this and without this idea about bias due to FCOIs is speculative. *Second*, the Open Payment database includes payments made in years 2015 and the list of editors is from 2016. We reviewed the journals’ websites and contacted them when necessary to confirm if the current editors also served as editors in 2014–15. However, some discrepancy is possible. *Third*, Open Payments database may not be accurate. Although physicians can dispute the records, very few physicians actually do so [38].

In conclusion, a significant number of editors of biomedical journals have financial conflicts of interest and very few are publicly disclosed. Specialty journal editors have more conflicts of interest compared to general medicine journal editors. Based on these results, guidelines should mandate specific, comprehensive and public disclosure of all COI for journal editors.

## Supporting information

S1 Dataset Contains the underlying data set and the individual data elements. (XLSX)
